# Why did people refuse vaccination during the pandemic? Exploring the impacts of trust and vaccine risk perception on COVID-19 vaccine hesitancy in China

**DOI:** 10.3389/fpubh.2025.1616129

**Published:** 2025-09-24

**Authors:** Dan Huang, Yi Wu, Hui Li

**Affiliations:** ^1^Department of Media and Communication, City University of Hong Kong, Hong Kong SAR, China; ^2^School of Media and Communication, Shenzhen University, Shenzhen, China

**Keywords:** vaccine hesitancy, trust, risk perception, self-efficacy, COVID-19, China

## Abstract

**Background:**

Vaccination was a critical step in combating the COVID-19 outbreak, but vaccine hesitancy was a prominent global concern in the pandemic. In China, the behavior of vaccination might be affected by the past vaccine-related scandals.

**Objective:**

This study investigated the factors contributing to vaccine hesitancy in China, with a focus on trust, vaccine risk perception, and self-efficacy. It aims to explore the predictors and mechanisms that influence vaccine hesitancy in China during the pandemic.

**Method:**

The study utilized a national survey fielded in 2021, with a representative sample of 3,000 Chinese adults. Quota sampling was employed to ensure regional and demographic representation of the sample. Key variables including institutional, media, and scientific trust, vaccine risk perception, and self-efficacy were measured adopting established scales from previous studies. A mediated moderation model was proposed. Trusts were hypothesized to affect vaccine hesitancy through vaccine risk perception. Moderation effect of self-efficacy on the relationship between vaccine risk perception and vaccine hesitancy was also proposed. Mediated moderation regressions were performed for model estimation.

**Results:**

Our analyses show that institutional trust was negatively associated with vaccine hesitancy [*b* = −0.41, *p* < 0.001, 95% CI (−0.47, −0.35)], while scientific trust and media trust was positively associated with vaccine hesitancy [*b* = 0.36, *p* < 0.001, 95% CI (0.32, 0.40); *b* = 0.21, *p* < 0.001, 95% CI (0.15, 0.27)]. Vaccine risk perception was also positively associated with vaccine hesitancy [*b* = 0.72, *p* < 0.001, 95% CI (0.68, 0.76)], with self-efficacy moderating the relationship. The relationship was more profound among those who had higher self efficacy [*b* = 0.29, *p* < 0.001, 95% CI (0.21, 0.37)]. Additionally, the mediating effects of vaccine risk perception were found.

**Conclusion:**

The findings revealed that trust in institutions significantly reduced vaccine hesitancy by lowering perceived risks. In contrast, media and scientific trust heightened vaccine risk perception and hesitancy. Additionally, the study demonstrated the role of self-efficacy in moderating these effects.

## Introduction

Effective vaccines for COVID-19 were a significant development that had the potential to reduce the risk of infection from the virus and mitigate the seriousness of the syndrome. Vaccination was a critical step in combating the COVID-19 outbreak (1). While a certain degree of vaccine hesitancy has been observed among the public for a variety of vaccines (1–3), The development and deployment of the COVID-19 vaccine elicited a notable level of public apprehension. A study that was conducted in 2020 during the pandemic found that approximately 25% of the American and 20% of the Canadian respondents were not willing to get vaccinated even if a COVID-19 vaccine were available (3). In the United Kingdom, 16.6% of respondents were very uncertain about COVID-19 vaccination, and 11.7% indicated strong hesitation (4). Similar reports have shown hesitancy regarding COVID-19 vaccines in other countries (2, 5–7).

In China, COVID-19 vaccine hesitancy was also present during the pandemic. Nearly half of those contacted indicated that they would wait to receive a vaccination until its safety was confirmed (2, 5). China is where the virus was initially identified, and the country has since implemented rigorous public health policies. Following the initial outbreak, the country enforced stringent containment measures; however, the subsequent relaxation of these policies led to widespread transmission across the nation (8). The lifting of the dramatic zero-COVID policy necessitated renewed vaccination drives, mainly targeting vulnerable populations such as older people, to address the challenges posed by new virus variants and ensure widespread immunity (9). However, despite the implementation of a national immunization program, concerns have been raised in China for a long time over the vaccine’s safety and efficacy (10).

Vaccine hesitancy can be defined as a delay in the acceptance or refusal of vaccines despite the availability of vaccination services (11). The 3Cs model proposed by the World Health Organization posits that vaccine hesitancy could be attributed to complacency, convenience, and confidence (12). Among the three factors, confidence appears to be the most salient in China in view of vaccine incidences in the past (13, 14). In this case, confidence refers to how safe people think a vaccine is. It is also related to a person’s confidence in the organizations and people in charge of developing and delivering immunization programs (15). Trust is one of the essential factors that influences vaccine confidence, and trust can be conceptualized with different dimensions (2). In this research, we focused on three facets of trust: institutional, media, and scientific trust.

Institutional trust refers to the public’s belief in the healthcare system’s capability and reliability. It plays a critical role in ensuring that people seek medical care, adhere to treatments, and accept health-related policies. Trust wanes when there are doubts regarding the healthcare system’s quality, openness, and ease of access, which are crucial for positive health outcomes (16). Gilson (17) and Freedman (18) underscore that trust is fundamental to how healthcare functions as a part of society.

Media trust refers to people’s trust in a variety of media outlets. The media historically serve as the primary vehicle for health intervention strategies, significantly influencing public health outcomes (19). Trust in the media is critical for the dissemination and acceptance of health-related information; it affects the extent to which the public adheres to health advisories (20) and engages in recommended health behaviors (21). During the COVID-19 infodemic, widespread misinformation and echo chambers—often reinforced by social media algorithms—undermined confidence in mainstream media and intensified vaccine hesitancy (22–24). Research on media trust during the pandemic further shows that these mechanisms systematically shaped perceptions of information credibility, amplifying doubts about vaccination (25).

Scientific trust refers to the public’s confidence in scientific knowledge, which has been deemed crucial for managing global challenges, such as the COVID-19 pandemic (26–28). Specifically, scientific trust impacts how citizens perceive crises and to what extent they accept related measures (29).

Recent studies have demonstrated that trust plays a complex role in shaping vaccine uptake. For example, the interplay between trust and COVID-19 information consumption was found to significantly influence vaccine and booster uptake (30), Similarly, patterns of news consumption and trust in public health leadership have been shown to shape COVID-19 knowledge and prejudice (31). Patterns of news consumption and trust in public health leadership have been shown to shape COVID-19 knowledge and prejudice (32). These findings suggest that the effects of trust are context-dependent, operating differently across populations and information environments.

Risk perceptions of vaccines is another psychological factor that influences vaccine hesitancy. In China, vaccine hesitancy, deeply influenced by the risk perceptions of vaccines, has posed major challenges to public health. Concerns over vaccine safety induced by past vaccine incidents negatively affect the success of vaccination programs, which are crucial for managing infectious diseases (33). The country’s unique socio-cultural and healthcare contexts require research specifically tailored to Chinese vaccine risk perceptions. However, empirical evidence from China can also provide valuable contributions to global efforts to address vaccine hesitancy (34, 35).

### Vaccine hesitancy and trust

Vaccine hesitancy, characterized as a delay in the acceptance or refusal to receive vaccines despite their availability, is influenced by safety concerns, complacency, and inconvenience (11). Trust is a crucial determinant in the decision-making process related to immunization. Public trust in vaccine safety and its influence on immunization refusal has been well documented, while distrust has been found to amplify perceived risks and contribute to increased hesitancy (36, 37). Additionally, Sinuraya et al. (38) highlighted that diminished confidence in authoritative institutions and media further exacerbates skepticism toward vaccines.

During health crises, trust in information sources has significantly influenced information-seeking behavior and the acceptance of health recommendations (39, 40). In addition, healthcare providers play a pivotal role in engendering trust in scientific and epidemiological evidence, thereby affecting vaccination uptake (41, 42).

Scientific trust, which is closely related to public confidence in scientific research regarding vaccine efficacy and safety, played a significant role during the pandemic. This concept affects the public due to their increasing exposure to scientific debates; its potential effect on public perception and health-related behavior has been highlighted often in the literature (43–45).

### Institutional trust

Trust in social institutions is based on beliefs in their capability to achieve desired goals effectively and in their commitment to act in the public’s best interests (46–48). As demonstrated in recent studies, institutions providing health information, such as health authorities and advisory bodies, have played a pivotal role in shaping vaccination intentions and behaviors (49).

There is a relationship between individuals’ willingness to be vaccinated and their level of trust in medical professionals, healthcare systems, and the pharmaceutical industry. Higher levels of trust generally indicate stronger intentions to be vaccinated, highlighting the importance of institutional trust in entities such as hospitals and public health agencies (50, 51). For example, it was found that a lower level of institutional trust led to negative attitudes toward vaccines during an Ebola outbreak (52). In Italy, greater trust in national healthcare institutions was found to predict a higher level of willingness to receive vaccines (53). In Saudi Arabia, the decision to receive a COVID-19 vaccine was found to be strongly influenced by trust in the healthcare system, which appeared to outweigh concerns over the virus’s perceived dangers (54).

Studies have shown that the relationships between healthcare providers and patients might account for the link between institutional trust and vaccination intention. Trust in doctors’ recommendations of vaccination facilitates positive doctor–patient communication and improves attitudes toward vaccination (55). Healthcare workers are often more trusted than other sources and, during the COVID-19 pandemic, were viewed as reliable sources of vaccine information (55–60).

Based on the discussion above, we hypothesized the following:

*H1*: A higher level of institutional trust will be associated with a lower level of vaccine hesitancy.

### Media trust

The media’s role as a primary source of health-related information was heightened during the COVID-19 pandemic. Garfin et al. (61) showed that the media acts as a crucial channel through which the public accesses information about health-protective behaviors. The unique position of mass media in communicating risks has been noted by Cottle (62) and Garfin et al. (61), emphasizing its influence in shaping public risk perceptions.

Social media platforms, such as Facebook and Twitter, have served as vital channels for disseminating disease-related information (63–65). However, the credibility of information on these platforms can be affected by personal relationships and trust dynamics within individual users’ networks (66–68). It is strengthened when positive opinion leaders endorse health messages, potentially increasing public trust in the media (69).

In contrast, the prevalence of misinformation in the media, especially social media, presents significant challenges to public health. Misleading content and conspiracy theories proliferating on these platforms have been found to be associated with increased vaccine hesitancy (70–72). The lack of regulatory oversight on social media can exacerbate this issue, leading to a heightened perception of risk regarding COVID-19 vaccines (72, 73). Given this issue, media trust may paradoxically lead to increased perceived vaccine risks and hesitancy.

Therefore, we asked the following:

*RQ1*: What are the effects of media trust on vaccine hesitancy?

### Scientific trust

Scientific trust refers to individuals’ confidence in scientific knowledge and expertise, especially during pandemics (56, 74, 75). Studies have shown that scientific trust is positively associated with adherence to pandemic measures and vaccination uptake (76–78). Moreover, trust in the scientific community has been identified as a decisive factor in encouraging individuals to vaccinate (51, 79).

In the context of a pandemic, cognitive trust—or confidence in scientific knowledge—is paramount (80). It involves a first-order scientific reasoning process through which individuals utilize scientific knowledge for problem solving and decision-making (81, 82). For example, the 2018 Wellcome Global Monitor survey revealed that a social consensus on science might foster a collective acknowledgment of the benefits of vaccination (83). General scientific knowledge is instrumental in acquiring specific virus-related knowledge and can influence risk perception and vaccine acceptance (84). Therefore, we can reasonably argue that individuals with higher scientific trust will be more engaged with vaccination information, thereby increasing their confidence in receiving vaccinations.

Based on the discussion above, we proposed the following:

*H2*: A higher level of scientific trust will be associated with a lower level of vaccine hesitancy.

### Vaccine risk perception and vaccine hesitancy

A key persistent risk is that people, communities, or society might be harmed at a certain time and location (85). Perceived risk refers to people’s subjective attitudes, beliefs, and judgments about potential dangers (86). The overall perception of individual health threats includes two dimensions: perceived severity and perceived susceptibility. The former refers to people’s subjective judgments of the severity of the consequences caused by health threats, while the latter refers to people’s judgments of the probability of being affected by these health threats (87).

In the current research, risk perception is focused on the perceived risk of the COVID-19 vaccine, emphasizing individuals’ subjective perceptions of the potential hazards associated with being vaccinated. Public vaccine hesitancy can be attributed to the potential risk associated with a vaccine (88, 89). The public’s confidence in the safety of vaccinations has been weakened by the frequent occurrence of vaccine scandals. Every year, there are many worldwide incidents of vaccine-induced diseases, disabilities, and even deaths, which increases people’s concerns about the safety of vaccines (90–92). For example, Changsheng Bio-technology, one of China’s largest vaccine producers, provided children with over 250,000 doses of an inferior pertussis vaccine in 2018. These inferior vaccines were manufactured using outdated ingredients accompanied by false batch numbers and manufacturing documents (93–95). This issue induced large-scale criticism of the vaccine industry in China. As a result, even vaccines that were not produced by the involved manufacturer were suspected with regard to their safety.

The case of the COVID-19 vaccines was very unique compared to other vaccines that had been previously used. Their development, clinical trials, and approval for use as an emergency public health product were conducted in a very short time, and there was seemingly insufficient proof of potential long-term effectiveness (96). Because of this, the public might be understandably skeptical about receiving a seemingly new vaccine in terms of a lack of assurance in terms of side effects.

One of the reasons for vaccine hesitancy is uncertainty about the vaccines used. Over the past 10 years, more than 10 vaccine crisis events have occurred in China, which has hurt public confidence in vaccines in general (2, 93, 94, 97, 98). In contrast, the danger presented by COVID-19 might have overshadowed the public’s worries about problematic vaccines (97), making people more likely to receive COVID-19 vaccination when it became available. Based on this discussion, we proposed the following:

*H3*: A higher level of vaccine risk perception will be associated with a higher level of vaccine hesitancy.

### Trust, vaccine risk perception, and vaccine hesitancy

Vaccine hesitancy may be correlated with several factors: the public’s trust in government and healthcare officials has been inconsistent; the legitimacy of science has faced scrutiny; social media platforms have enabled a wider dissemination of dramatic personal experiences; and significant public health and vaccine anxieties have been prevalent in the media (88, 99). These components, collectively, might have influenced individuals’ reluctance to vaccinate.

Trust in healthcare providers and authorities is foundational to the acceptance of medical interventions (17). A link between trust in vaccines and vaccination uptake has been documented, highlighting the mitigating effect of trust on vaccine hesitancy (51). Vaccine risk perception can be reasonably argued as exerting a mediating role in this relationship. Individuals with higher trust in vaccine benefits and safety tended to perceive lower risks associated with vaccines, which increased the rate of vaccine acceptance (100). In contrast, those with diminished trust are prone to perceive higher risks, leading to increased hesitancy (88). The importance of risk perception is further emphasized when a new vaccine is developed. During the initial rollout of COVID-19 vaccines, risk perception was a critical determinant of public willingness to receive the vaccine (59). The interplay among media trust, vaccine risk perception, and vaccine hesitancy was identified as crucial for understanding public health compliance, particularly during global health emergencies such as the COVID-19 pandemic. Media trust significantly influenced public attitudes toward vaccines, as individuals commonly sought health information from media sources (51). Credible media outlets are likely to enhance vaccine uptake by reducing the perceived risks associated with vaccines (101). Conversely, mistrust in the media was found to intensify vaccine hesitancy by amplifying perceived risks (102).

While scientific trust typically fosters compliance with health measures, paradoxically, it may also lead to heightened vaccine risk perceptions due to the critical evaluation of scientific communications and potential side effects. Therefore, individuals’ exposure to scientific information must also consider how that information is perceived and used; it can manifest in proactive engagement with health behaviors and increased vigilance regarding health interventions. Beck’s concept of reflexive conscientization articulates this dynamic, suggesting that informed skepticism is a feature of contemporary attitudes toward science, including pandemic and vaccine safety (103, 104). Following this logic, we argue that individuals with higher scientific trust may be more engaged with vaccination information. They may also exhibit a higher risk perception of a vaccine due to their critical evaluation of scientific claims and concerns about vaccines, thereby increasing vaccine hesitancy.

Based on the discussion above, we propose the following:

*H4a*: Vaccine risk perception will mediate the relationship between institutional trust and vaccine hesitancy.

*H4b*: Vaccine risk perception will mediate the relationship between media trust and vaccine hesitancy.

*H4c*: Vaccine risk perception will mediate the relationship between scientific trust and vaccine hesitancy.

### The role of self-efficacy

Self-efficacy is defined as an individual’s belief in their capabilities to execute behaviors necessary to achieve specific goals (105). In the context of health communication, self-efficacy plays a pivotal role in influencing health behaviors, including vaccination decisions (106). It pertains to an individual’s belief in their capacity to modify habits and manage their functions when encountering challenges (107). Research has consistently shown that individuals with higher levels of self-efficacy are more likely to engage in health-promoting behaviors (108).

In the health belief model (HBM), self-efficacy is a critical factor in comprehending and tackling vaccine hesitancy. The HBM proposes that health behaviors are influenced by personal beliefs about health threats, the perceived benefits of and barriers to action, and cues to action (109, 110). Self-efficacy—the belief in one’s ability to perform a behavior—is found to moderate these influences, especially in the context of vaccine hesitancy (108, 111).

Past research has shown that self-efficacy independently predicts not only the intention to vaccinate (112) but also actual vaccination behavior (113, 114). A Hong Kong survey on the human papilloma virus (HPV) vaccination revealed that self-efficacy significantly influenced the uptake of the vaccine among physicians and nurses. It was determined that self-efficacy was the sole significant cognitive and background variable correlated with the acceptance of the HPV vaccine by these healthcare professionals (115). Intervention strategies targeted at increasing self-efficacy have been confirmed to be successful in fostering vaccine acceptance (116). Health communications that inform individuals with the necessary skills and resources increase confidence in being vaccinated, consequently reducing hesitancy (117). Interventions that contained components designed to enhance self-efficacy, such as skill-building activities and empowerment strategies, were more effective at reducing vaccine hesitancy (118). These findings suggest that self-efficacy might be a potential factor that interacts with other predictors within the HBM framework. Therefore, we proposed the following:

*H5*: Self-efficacy will moderate the relationship between vaccine risk perception and vaccine hesitancy.

**Figure 1:**
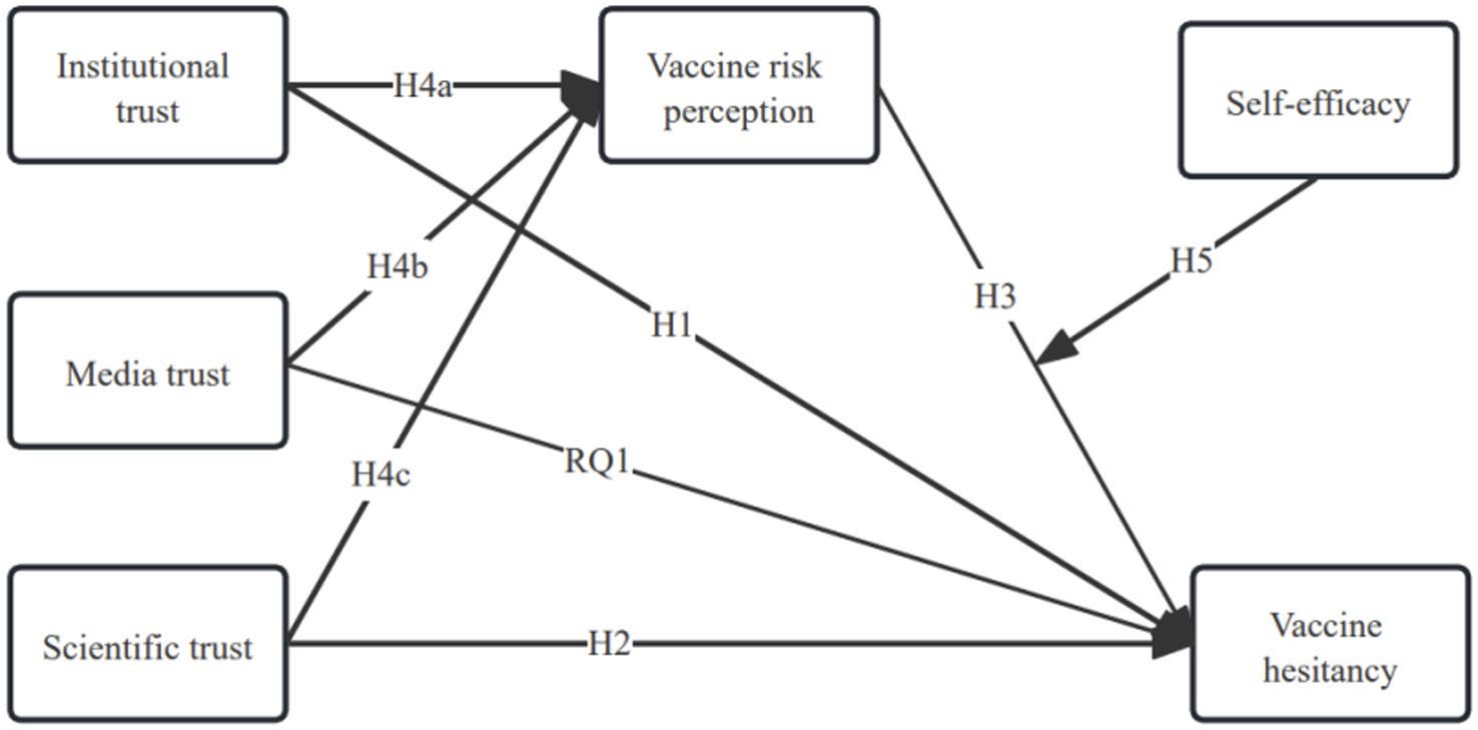
Theoretical model of the study.

Integrating the hypothesized relationships, a mediated moderation model was proposed. The theoretical model is shown in Figure 1.

## Materials and methods

### Sample

The study was conducted in China from May 31 to July 10, 2021. During that period, the Chinese government deployed a zero-COVID policy nationally. Cross-sectional data were collected through a survey commissioned to a commercial survey research company. To achieve a representative sample, a quota sampling method was employed. The most up-to-date CNNIC (China Internet Network Information Center) report was used to establish the quotas for group subcategories of gender, age, and education (119). The final sample comprised 3,000 Chinese citizens aged 18 and older, with a response rate of 31.96%, calculated following the American Association for Public Opinion Research (AAPOR) standard definition RR1 (120). Although modest, the response rate was acceptable compared to other large-scale online surveys. Quota sampling was used to mitigate potential non-response bias and improve representativeness. The demographic breakdown was 51.03% male and 48.97% female, with ages ranging from 18 to over 60. Approximately 75.5% of respondents reported a monthly household income between 6,000 and 30,000 yuan, and 67.93% held a college degree or higher. Participants were drawn from all major regions of mainland China (East, Central, and West). Urban residents were slightly overrepresented, reflecting the demographic profile of China’s internet users. The skewness toward urban and highly educated participants should be considered when interpreting generalizability of the results. It was further addressed in the Limitations section. The demographic characteristics of the sample are shown in Table 1. The study was approved by the Institutional Review Board of Shenzhen University.

**Table 1:** Sample demographics (*N* = 3,000).

Variables	Frequency	Percentage (%)
Gender		
Male	1,531	51.03
Female	1,469	48.97
Age		
18–29	641	21.37
30–39	737	24.57
40–49	676	22.53
50–59	543	18.10
60 and above	403	13.43
Education		
Elementary school and below	55	1.83
Junior high school graduation	275	9.17
Graduated from high school	634	21.13
College graduation	786	26.20
Bachelor’s degree and above	1,250	41.67
Family income(average/month)		
Less than 1,000 RMB (including 1,000)	17	0.57
1,001–3,000 RMB	48	1.60
3,001–6,000 RMB	355	11.83
6,001–10,000 RMB	875	29.17
10,001–30,000 RMB	1,390	46.33
30,001–60,000 RMB	272	9.07
60,001–100,000 RMB	35	1.17
Above 100,000 RMB	8	0.27

### Measures

#### Control variables

Prior studies have demonstrated that certain demographic factors, including gender, age, education, and income, influence a wide range of behaviors that could be related to the health behaviors examined in this study. Thus, to avoid the potential confounding effects of these variables, we included them as controls in our analysis.

#### Media trust

Media trust was measured using a five-point Likert scale (1 = completely distrust, 5 = completely trust). Respondents were asked about their trust in central government media, local media, commercial media, social media platforms, search engines, bulletin board systems (BBSs), video apps, and prominent bloggers. These categories reflect the most prominent information channels in China’s media environment. The items were designed based on prior comparative studies of media trust (121, 122) but adapted to the Chinese context.

#### Institutional trust

Institutional trust was measured using a five-point Likert scale (1 = completely distrust, 5 = completely trust). Respondents indicated their trust in the central government, local governments, police departments, and educational institutions. The measurement draws conceptually from established frameworks of political and institutional trust (123, 124), while tailoring the items to the Chinese governance system.

#### Scientific trust

Scientific trust was measured using a five-point Likert scale (1 = completely distrust, 5 = completely trust). Respondents were asked about their trust in the knowledge conveyed by scientific researchers and the role of science in different settings. Items were adapted from established surveys on public attitudes toward science, including the U. S. National Science Board’s Science and Engineering Indicators and the European Commission’s Eurobarometer on Science and Technology, which have been widely used to assess trust in science and perceptions of scientists (125, 126). The wording was slightly modified to fit the context of COVID-19.

#### Vaccine risk perception

Vaccine risk perception was measured using a four-point scale (1 = definitely wrong, 4 = definitely right). Respondents evaluated statements such as “getting vaccinated can cause COVID-19 infection,” “the vaccine’s effectiveness lasts for only a short period, therefore, there is no need for it,” and “people who receive domestic vaccines are likely to experience significant side effects.” These items were adapted from the WHO Vaccine Hesitancy Survey Module (127) and the Vaccine Confidence Project (128), which emphasize concerns about safety, efficacy, and necessity.

#### Self-efficacy

Self-efficacy was measured using a five-point Likert scale (1 = strongly disagree, 5 = strongly agree). Respondents evaluated statements including “I will encourage those who are close to me to get COVID-19 vaccinations,” “I am very concerned about the COVID-19 vaccine information,” “I believe I have received sufficient information about the COVID-19 vaccine,” and “I will express my opinion about the COVID-19 vaccine on the internet.” These items were adapted based on the Health Belief Model self-efficacy framework (129) and revised to reflect vaccination-related behaviors in the Chinese context.

#### Vaccine hesitancy

Vaccine hesitancy was measured using a five-point Likert scale (1 = strongly disagree, 5 = strongly agree). Respondents evaluated statements such as “I worry about the side effects of receiving the COVID-19 vaccine,” “I suspect the effectiveness of the COVID-19 vaccine,” “I am concerned about the safety of the COVID-19 vaccine,” and “I lost my confidence with the COVID-19 vaccine due to previous vaccine-related experiences.” These items were adopted from the WHO Vaccine Hesitancy Survey Module (127) on vaccine hesitancy and aligned with the “confidence” dimension of the 5C model (100), with further adjustments to reflect Chinese vaccine scandals.

All the multi-item constructs showed acceptable internal consistency (Cronbach’s *α* > 0.70). Forward–backward translation procedures ensured accuracy of the Chinese version, and a pilot test with a small group of Chinese respondents confirmed item clarity and cultural relevance.

### Data analysis

Data quality was ensured by screening for incomplete or inconsistent responses. Any missing data were handled using listwise deletion, as the proportion of missing responses was low (<2% for key variables). Sensitivity checks confirmed that the demographic profile of the retained sample did not significantly differ from the original before deletion, and all analyses were conducted on the cleaned dataset.

Data analysis was performed using SPSS version 26 and PROCESS macro version 4.1 (130). A descriptive statistical analysis and bivariate correlation analysis were first conducted, followed by estimation of the main effect model. In the second step, the mediation effect was tested using PROCESS Model 4. Finally, we tested the mediated moderation model was examined using PROCESS Model 14. Continuous variables were mean-centered before the analysis, and bootstrapping 5,000 samples with 95% confidence intervals was performed.

## Results

### Descriptive statistics and correlations

We first performed a descriptive analysis of the main variables. The results revealed that respondents had a moderate level of trust in the media (*M* = 3.44, SD = 0.65, Cronbach’s *α* = 0.89), while institutional trust was relatively high (*M* = 4.16, SD = 0.47, Cronbach’s *α* = 0.73). Trust in the central government was rated the highest, with 67.6% of participants assigning it the highest score of 5. Scientific trust was at a moderate level (*M* = 3.00, SD = 0.85, Cronbach’s *α* = 0.79). The level of vaccine risk perception was also moderate (*M* = 1.75, SD = 0.68, Cronbach’s *α* = 0.88). Approximately 46.8% of the respondents firmly rejected the idea that recipients of domestic vaccines were at a high risk of side effects.

The level of self-efficacy was generally positive (*M* = 3.95, SD = 0.55, Cronbach’s *α* = 0.75). Over 86% of the respondents agreed or strongly agreed with encouraging vaccination, and approximately 79% were proactive in seeking and sharing vaccine information.

According to our scale, the level of vaccine hesitancy was moderate (*M* = 2.71, SD = 0.93, Cronbach’s *α* = 0.89). Respondents expressed the most concern about the side effects, efficacy, and safety of the vaccines. In addition, there were concerns about economic factors, fears of counterfeit vaccines, and past negative experiences.

The Pearson correlation analysis revealed significant correlations between the independent variables (institutional trust, media trust, and scientific trust) and the dependent variable (vaccine hesitancy). Institutional trust showed a weakly negative correlation (*r* = −0.19), while media trust and scientific trust exhibited positive correlations (*r* = 0.24) and (*r* = 0.40), respectively, with vaccine hesitancy. Vaccine risk perception and vaccine hesitancy showed a strong correlation (*r* = 0.62). Since all the correlation coefficients fell below the 0.7 threshold, collinearity could be excluded for the variables studied (see Table 2).

**Table 2:** Pearson correlation coefficients between variables (*N* = 3,000).

No.	Variable	1	2	3	4	5	6	7	8	9	10
1	Age	—									
2	Gender	0.00	—								
3	Education	−0.60***	−0.03	—							
4	Family income	−0.21***	0.00	0.39***	—						
5	Institutional trust	−0.16***	0.04	0.16***	0.10***	—					
6	Media trust	0.23***	−0.02	−0.06**	0.09***	0.26***	—				
7	Scientific trust	0.23***	−0.04*	−0.13***	0.05*	0.00	0.44***	—			
8	Vaccine risk perception	0.23***	−0.06**	−0.13***	−0.04*	−0.19***	0.36***	0.49***	—		
9	Self-efficacy	0.00	0.00	0.11***	0.16***	0.40***	0.43***	0.18***	0.01	—	
10	Vaccine hesitancy	0.21***	−0.02	−0.14***	−0.07***	−0.19***	0.24***	0.40***	0.62***	−0.04*	—
	M	4.78	1.49	4.95	4.52	4.16	3.44	3.00	1.75	3.95	2.71
	SD	—	—	—	—	0.47	0.65	0.85	0.68	0.55	0.93

*p < 0.05; **p < 0.01; ***p < 0.001.

### Hypothesis testing

H1 posited that a higher level of institutional trust would be associated with a lower level of vaccine hesitancy. As shown in Table 3, there was a significant negative association between institutional trust and vaccine hesitancy [Model 1; b = −0.41, *p* < 0.001, 95% CI (−0.47, −0.35)], supporting H1.

**Table 3:** Regression analysis of the main effect, mediation effect, and mediated moderation effect on vaccine hesitancy (*N* = 3,000).

Variable	Model 1	Model 2	Model 3
Constant	2.74***(0.20)	1.72***(0.18)	2.96***(0.20)
Control variable			
Age	0.04*(0.01)	0.01(0.01)	0.02(0.01)
Gender	0.00(0.03)	0.03(0.03)	0.03(0.03)
Education	−0.00(0.02)	−0.02(0.02)	−0.02(0.02)
Family income	−0.06***(0.02)	0.00(0.00)	−0.03(0.01)
Independent variable			
Institutional trust	−0.41***(0.03)	−0.15***(0.03)	−0.17***(0.03)
Media trust	0.21***(0.03)	0.01(0.03)	0.04(0.03)
Scientific trust	0.36***(0.02)	0.15***(0.02)	0.14***(0.02)
Mediator			
Vaccine risk perception		0.72***(0.02)	0.68***(0.02)
Moderator			
Self-efficacy			−0.04(0.03)
Vaccine risk perception*Self-efficacy			0.29***(0.04)
Adjusted *R*^2^	0.22	0.41	0.42
*F* value	(7,2,992) = 121.88***	(8,2,991) = 255.64***	(10,2,989) = 214.00***

Entries are standardized coefficients, standard errors in parentheses. *p < 0.05; **p < 0.01; ***p < 0.001.

RQ1 inquired about the impact of media trust on vaccine hesitancy. A positive association between media trust and vaccine hesitancy was observed [Model 1; *b* = 0.21, *p* < 0.001, 95% CI (0.15, 0.27)], indicating that a higher level of media trust would lead to a higher level of vaccine hesitancy.

H2 proposed that a negative association existed between scientific trust and vaccine hesitancy. As the results show, scientific trust was positively related to vaccine hesitancy [Model 1; *b* = 0.36, *p* < 0.001, 95% CI (0.32, 0.40)], indicating that a higher level of scientific trust was linked to a higher level of vaccine hesitancy; thus, H2 was rejected.

H3 proposed that there was a positive association between vaccine risk perception and vaccine hesitancy. Vaccine risk perception was positively associated with vaccine hesitancy [Model 2; *b* = 0.72, *p* < 0.001, 95% CI (0.68, 0.76)], supporting H3.

H4a–H4c proposed that vaccine risk perception would mediate the relationships between the trust variables and vaccine hesitancy. The indirect effects of institutional, media, and scientific trust on vaccine hesitancy via vaccine risk perception were examined using Model 4 of the PROCESS Macro. The bootstrapping method (with 5,000 resamples) yielded 95% confidence intervals for indirect effects that did not include zero (institutional trust: −0.29, −0.23; media trust: 0.17, 0.22; scientific trust: 0.19, 0.24). These results indicated that vaccine risk perception served as a mediator in the relationships between each trust variable and vaccine hesitancy, thus supporting H4a–H4c. Although significant, the effect sizes were modest, accounting for roughly 10–15% of the variance in hesitancy outcomes, which is consistent with effect sizes reported in prior health communication research. It suggests that while risk perception is an important mechanism, other psychological or contextual factors possibly exist to account for the mediated paths.

**Figure 2:**
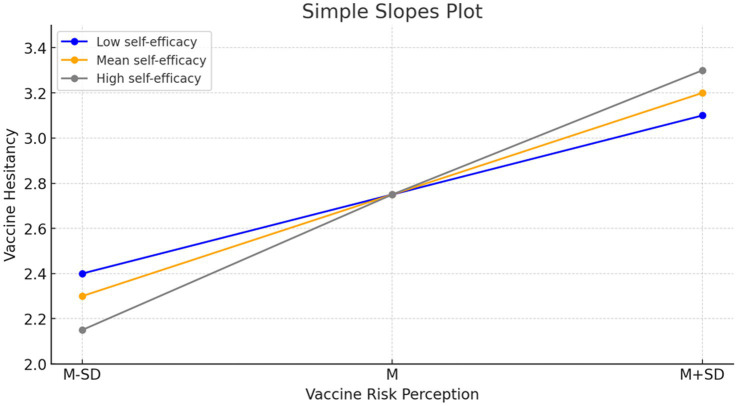
Vaccine hesitancy predicted by vaccine risk perception moderated by self-efficacy.

H5 proposed that self-efficacy moderated the relationship between vaccine risk perception and vaccine hesitancy. We found that the moderating effect was significant [Model 3; *b* = 0.29, *p* < 0.001, 95% CI (0.21, 0.37)]. The conditional effect was evidenced in that the 95% confidence intervals from the PROCESS Macro Model 14 analysis did not encompass zero (*M* – 1 SD: 0.45, 0.59; M: 0.63, 0.72; M + 1 SD: 0.78, 0.89). Specifically, the impact of vaccine risk perception on vaccine hesitancy was more intense among individuals with low self-efficacy. In contrast, individuals with high self-efficacy demonstrated low vaccine hesitancy at a lower risk perception level, while increased perceived risk significantly amplified hesitancy among these individuals (see Figure 2). H5 was supported.

**Figure 3:**
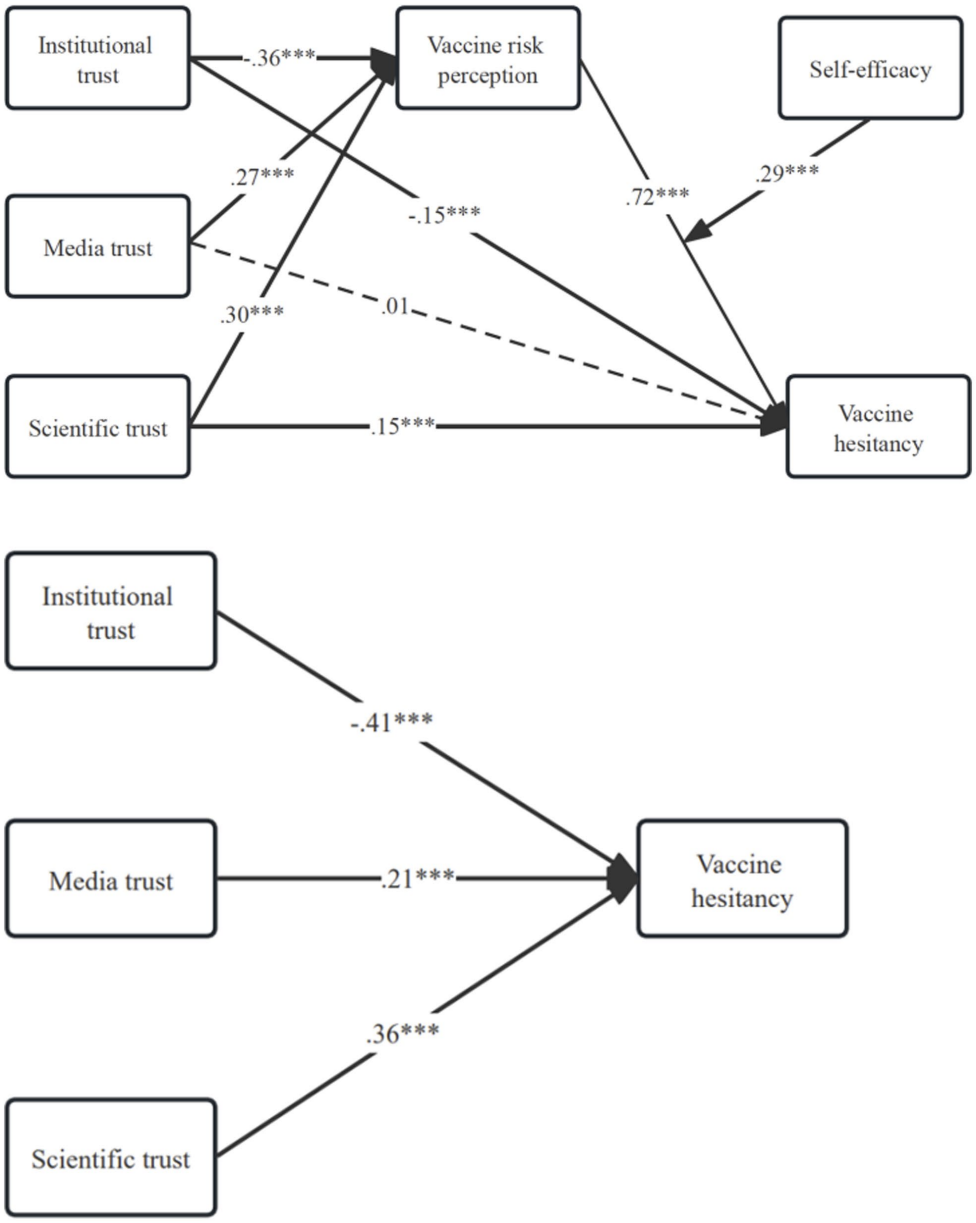
Evaluation outcomes of the mediated moderation model.

The analysis revealed that self-efficacy moderated the relationship between trust and vaccine hesitancy. A negative indirect effect of institutional trust on vaccine hesitancy was observed; it became more intense with increasing self-efficacy (−0.19 at M–1SD; −0.24 at M; −0.30 at M + 1SD). The coefficients at one standard deviation below the mean, at the mean, and at one standard deviation above the mean were −0.19, −0.24, and −0.30, respectively. In contrast, media trust and scientific trust were positively associated with vaccine hesitancy. This association was strengthened at higher levels of self-efficacy, with effect sizes escalating from 0.14 to 0.22, and 0.16 to 0.25. These nuanced findings underscore the multifaceted interplay among trust, self-efficacy, and risk perception in the context of vaccine hesitancy, suggesting that the nature of trust and the level of self-efficacy coalesce to shape perceptions of vaccine risk and the intensity of hesitancy (see Table 4). The overall evaluations are illustrated in Figure 3.

**Table 4:** Conditional indirect effects of trust variables on vaccine hesitancy moderated by self-efficacy.

Variable	Self-efficacy	Effect	Boot SE	Boot 95% CI
Institutional trust	M-1SD	−0.19	0.02	[−0.22, −0.16]
	M	−0.24	0.02	[−0.28, −0.21]
	M + 1SD	−0.30	0.02	[−0.34, −0.26]
Media trust	M-1SD	0.14	0.01	[0.11, 0.17]
	M	0.18	0.01	[0.16, 0.21]
	M + 1SD	0.22	0.02	[0.19, 0.26]
Scientific trust	M-1SD	0.16	0.01	[0.13, 0.18]
	M	0.20	0.01	[0.18, 0.23]
	M + 1SD	0.25	0.01	[0.22, 0.28]

Taken together, the mediated moderation analysis showed that the indirect effects of institutional, media, and scientific trust on vaccine hesitancy via risk perception were consistently significant, with effect sizes ranging from small to moderate (|b| = 0.14–0.30). Importantly, these indirect effects were contingent on self-efficacy levels, as visualized in Figure 3: higher self-efficacy amplified both the protective role of institutional trust and the risk-enhancing effects of media and scientific trust. It shows a clear interpretation of how trust and self-efficacy jointly shape vaccine hesitancy.

## Discussion

The present study aimed to investigate the relationships among trust in institutions, media, and science; vaccine risk perception; self-efficacy; and vaccine hesitancy. The findings of the study provide insights into the factors that contribute to vaccine hesitancy and highlight the importance of vaccine risk perception and self-efficacy in the context of China, where a zero-COVID policy was deployed.

First, we found that institutional trust had a negative impact on vaccine risk perception and vaccine hesitancy. It is supported by recent evidence showing that individuals with lower institutional trust are more likely to exhibit vaccine hesitancy (131, 132). Similarly, Murphy et al. found that mistrust in authoritative information sources is linked to higher level of COVID-19 vaccine hesitancy in the UK and Ireland (133). Trust in the Chinese government and health authorities reduced hesitancy to obtain the COVID-19 vaccine. This suggests that although there were vaccine scandals in China in the past, people generally still had faith and confidence that the government and health authorities would supply a safe and effective COVID-19 vaccine. Interestingly, trust in science was associated with higher perceived vaccine risk and hesitancy. One explanation is that individuals who strongly trust science also tend to be more scientifically literate and thus more sensitive to uncertainties regarding the rapid development of COVID-19 vaccines. Prior research has shown that risk perception and health behaviors are not always linearly related—greater awareness of risks can sometimes reduce uptake (134). Another explanation is the “double-edged sword” of transparent communication: individuals with higher trust level may take scientific warnings about side effects or incomplete data more seriously, which can heighten caution (135). In the Chinese context, scientific trust often overlaps with reliance on official expert communication, so when messages emphasized uncertainties, those with higher trust were more likely to perceive greater risks (136). Trust in the media induced individuals to perceive that the COVID-19 vaccine was unsafe. Continuous arguments and discussions over vaccine issues during the pandemic period might have had a serious negative impact on citizens’ attitudes. Prior studies confirm that exposure to misinformation and conflicting information substantially reduces vaccination intent (137), and social media further amplified this effect, with widespread false claims contributing to hesitancy (138). In China’s media environment, censorship and selective exposure also played a role: while strict information control limited the spread of rumors, it also reduced transparency, potentially leading some individuals to question official narratives (139, 140). The factors—misinformation, censorship, and selective exposure—jointly shaped how citizens interpreted vaccine safety. Despite advocacy from state governments to promote vaccination, the amount of misinformation, conspiracy theories, and other disorienting types of information was overwhelming on social media, which might have negatively impacted attitudes toward the COVID-19 vaccine.

Second, in the study, we were able to conceptually describe how trust and skepticism influenced public health decisions in the very specific context of the epidemic in China. While having faith in health institutions was beneficial for the public’s desire to be vaccinated, self-efficacy was another critical factor that affected the process of health decision-making. Public health professionals should build trust and encourage people to be well informed and think critically. We found that the public was cautious due to the rapid development of the COVID-19 vaccine, suggesting that trust in science might even increase the skepticism of the public (as they possibly felt proper scientific procedures were not being followed). Cross-national surveys indicate that China had one of the highest estimated vaccine acceptance rates—nearly 90%—among 19 countries studied (141), and domestic data suggest that transparent communication, medical authority endorsements, and vaccination convenience significantly influence public acceptance within China (10). Scientific information, including transparent communication strategies explaining the scientific process and rigorous safety checks for vaccine development, might have provided more objective views on the safety and risks of the COVID-19 vaccine. People who make health decisions based on scientific information might have more concerns, especially about the unknown long-term effects of a new vaccine.

Third, the mediation effect of risk perception between trust in various entities (governmental institutions, media, and the scientific community) and vaccine hesitancy, as found in the study, suggests that fostering trust can attenuate hesitancy by influencing how the public perceives vaccine risks. The positive correlation between vaccine risk perception and hesitancy underscores the challenges posed by historical vaccine scandals, which have indelibly marked collective consciousness. The vaccine related incidents have led to measurable delays in vaccination schedules, as documented after the Changchun Changsheng incident (142). However, the Chinese government’s robust response to the COVID-19 pandemic, which prioritized transparency and safety, played a crucial role in reshaping these perceptions. This was evident during the pandemic when the government’s efforts to communicate effectively about the safety and efficacy of COVID-19 vaccines were instrumental in managing the public’s apprehension. Although transparent communication of vaccine risks may transiently affect uptake, it has been shown to build trust over time (135). Moreover, the psychological impact of compulsory vaccination warrants closer attention: while mandates can rapidly increase coverage, they may also provoke psychological reactance and reduce long-term trust in health authorities (143, 144). The dual effects highlight the need to balance public health goals with respect for individual autonomy, particularly in nations where collective responsibility is highly emphasized. In addition, the role of self-efficacy in moderating the relationship between vaccine risk perception and hesitancy is particularly notable. The results demonstrate that individuals with higher self-efficacy exhibited a more discerning response to vaccine risks, which can either diminish or amplify vaccine hesitancy. The Chinese government introduced mandatory vaccination policies during the pandemic. This policy aimed to achieve high vaccination rates rapidly to control the spread of the virus. While compulsory vaccination can ensure wide-range vaccine uptake, it can also provoke resistance among those who prioritize personal choice or have concerns about vaccine safety.

## Limitations and conclusions

The study has a few limitations. First, the sample underrepresents older adults (only 13.4% were aged 60 or above), a key high-risk group, as well as rural residents. Generalization of the results should be made with caution. Second, the response rate was not high (31.96%), although acceptable, it raises the possibility of non-response bias (120). Participants who chose to respond may differ systematically from non-respondents—for instance, being more health-conscious or digitally active. Although quota sampling based on demographic benchmarks improved balance, the bias cannot be ruled out. Third, the measure did not distinguish between sub-dimensions of institutional trust (e.g., healthcare v.s. central government), which may exert distinct effects. Fourth, the survey data were collected during China’s “zero-COVID” policy period amid an active vaccination campaign, so the findings cannot fully extend to other contexts or time periods. Last but not least, the cross-sectional and self-reported design precludes causal inference and may be subject to social desirability bias. Despite these caveats, the study provides timely insights that should be interpreted with appropriate caution.

In conclusion, the study elucidates the dynamics among trust, vaccine hesitancy, vaccine risk perception, and self-efficacy. The findings revealed that trust in institutions significantly reduced vaccine hesitancy by lowering perceived risks. In contrast, media and scientific trust heightened vaccine risk perception and hesitancy. Additionally, the study demonstrated the role of self-efficacy in moderating these effects. This study identified several factors and mechanisms that contribute to vaccine hesitancy and provides insights for dealing with this important public health issue during times of crisis.

## Data Availability

The raw data supporting the conclusions of this article will be made available by the authors, without undue reservation.
